# Identification of lobetyolin as a major antimalarial constituent of the roots of *Lobelia giberroa* Hemsl

**DOI:** 10.1016/j.ijpddr.2022.01.002

**Published:** 2022-01-22

**Authors:** Getnet Tadege, Yonatan Alebachew, Ariaya Hymete, Solomon Tadesse

**Affiliations:** Department of Pharmaceutical Chemistry and Pharmacognosy, School of Pharmacy, College of Health Sciences, Addis Ababa University, Addis Ababa, Ethiopia

**Keywords:** Antimalarial activity, *Lobelia giberroa*, Lobetyolin, *Plasmodium berghei*, Polyacetylene, Traditional medicine

## Abstract

*Lobelia giberroa Hemsl.* is an endogenous Ethiopian medicinal plant with a long history of use in the treatment of malaria, bacterial and fungal diseases, and cancer. Here, we present the *in vivo* bioassay-guided fractionation of the 80% methanol extract of *L. giberroa* roots, which led to the isolation of lobetyolin. *L. giberroa* roots were extracted with 80% methanol, and the dried 80% methanol extract was fractionated with hexane, ethyl acetate, methanol, and water. Acute oral toxicity study was conducted according to the Organisation for Economic Co-operation and Development Guideline 425 by using female Swiss albino mice. Antimalarial activity was assessed in *Plasmodium berghei*-infected Swiss albino mice. Through *in vivo* bioassay-guided fractionation processes lobetyolin, a C14-polyacetylene glucoside, was isolated from the methanol fraction by silica gel column chromatography as the main active ingredient from the plant. The chemical structure of lobetyolin was elucidated by interpretation of spectroscopic data (^1^HNMR, ^13^CNMR, IR. MS) including two dimensional NMR. The plant extract was considered safe for administration up to 2000 mg/kg. In the four-day suppressive test, the 80% methanol extract (400 mg/kg), methanol fraction (400 mg/kg), and lobetyolin (100 mg/kg) exhibited antimalarial activity, with chemosuppression values of 73.05, 64.37, and 68.21%, respectively. Compared to the negative control, which had a mean survival time of 7 days, the lobetyolin (100 mg/kg) and methanol fraction (400 mg/kg) treated groups had mean survival times of 18 and 19 days, respectively. The current study supports the traditional use of the plant for the treatment of malaria. The structural differences between lobetyolin and existing antimalarials, as well as its previously unknown antimalarial activity, make it of interest as an early lead compound for further chemical optimization.

## Highlights

• Multi-drug resistant malaria parasites urge the discovery of newer drugs.
• *Lobelia giberroa* is an indigenous traditional antimalarial plant of Ethiopia.
• *In vivo* antimalarial-activity-guided fractionation led to the isolation of lobetyolin.
• Lobetyolin exhibits a promising antimalarial activity with an ED_50_ of 36.8 mg/kg.
• Lobetyolin is a potential lead compound to develop a new class of antimalarial drugs.

## Graphical abstract

**Figure:**
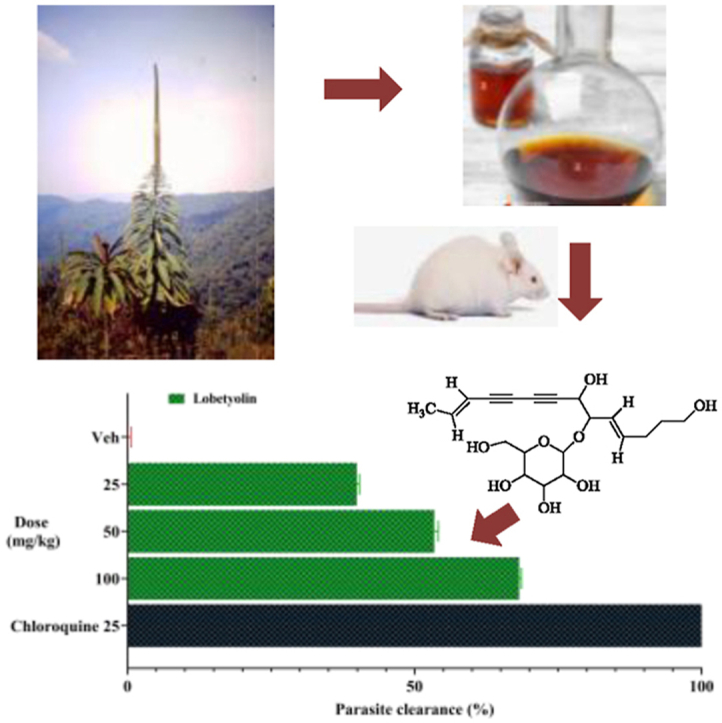

## 1 Introduction

Globally, there were an estimated 241 million malaria cases and 627 thousand deaths in 2020 (WHO, 2021). Ninety-six percent of these deaths occur in Africa, particularly in sub-Saharan Africa. In Ethiopia, the disease counts for 7% of outpatient visits to health clinics and is the third largest cause of morbidity (Girum et al., 2019). Frequently, pregnant women and children under five years old are the ones severely attacked by the disease (Snow et al., 2017)*.*

In addition to shortage of preventive tools and chemotherapeutic drugs in malaria endemic regions, the major cause of this alarming morbidity and mortality is the emergence of multi-drug resistant strains of malaria parasites (Uwimana et al., 2020). To address the challenges of drug resistance, there is a need for the development of safe and efficacious antimalarial drugs. One of the most important tactics in this regard is to explore new chemical entities derived from natural resources (Mennai et al., 2021; Tostes et al., 2021).

*Lobelia* (Campanulaceae, Lobelioideae subfamily) is a flowering plant genus of 415 species (Folquitto et al., 2019). The genus drew the attention of phytochemists mainly due to the discovery of bioactive piperidine alkaloids such as lobinaline and lobeline in the early 1950s (Zheng et al., 2021). Subsequent phytochemical researches of *Lobelia* led to the discovery of several novel bioactive secondary metabolites including flavonoids, terpenoids, polyacetylenes, and coumarins. *Lobelia* species have long been used to cure a variety of ailments, and several species possess genuine antimicrobial, anti-inflammatory, cytotoxic, and anticonvulsant activities (Folquitto et al., 2019).

*L. giberroa* is indigenous to Ethiopia, and is known by a variety of names including Jibera (Amharic), Maranga (Oromifan), Shambato (Kefa) (Abate, 1989). In Ethiopia, the roots of *L. giberroa* are used to treat malaria and eye disorders (Chekole, 2017). However, there are no reports regarding the biological activity or phytochemistry the plant. The aim of this study was, therefore, to determine the antimalarial activity of the roots of *L. giberroa* against *Plasmodium berghei* infection in mice and to identify the main active molecule/s.

## 2 Methods

### 2.1 Chemicals and instruments

Hexanes, chloroform, ethyl acetate (all from Sigma-Aldrich Co., MO, USA), methanol (Carlo Erba, France) and distilled water (AAU laboratory) were the solvents used for extraction, fractionation and isolation. Chromatographic separation was performed by analytical TLC on Silica gel 60 F254 (0.2 mm thick), column chromatography on Silica gel 60 (70–240 mesh) (Merck KGaA, Darmstadt, Germany) and preparative TLC on silica gel HF 254 (500 g; LOBACHEMIE, India), (20 cm × 20 cm) with thickness of 0.50 mm. The following chemicals and drugs were used during *in vivo* antimalarial activity test: Trisodium citrate (BDH Chemicals Ltd, England), Geimsa (ESJAY Chemicals, Maharashtra, India), Chloroquine phosphate (EPHARM, Ethiopia) and Tween 80 (UNI-CHEM chemical reagents, India).

^1^H and ^13^C NMR spectrum were recorded on Bruker Avance DMx400 FT-NMR spectrometer using TMS as internal standard, at the Department of Chemistry, Collage of Natural Sciences, Addis Ababa University, Ethiopia. Mass spectrometry was performed using an Agilent 1100 series system (Agilent system, USA), and ionization of sample was carried out using ESI-API (capillary voltage, 4000 V; fragmentor, 160 V; drying gas temperature, 350 °C; gas flow (N_2_), 10 l/min; nebulizer pressure, 50 psig). The purity of the isolated compound (1 mg/ml in methanol) was determined by LC/MS system with diode array detector (DAD) using the following instrumentation and analytical conditions: an Agilent 1100 series LC/MS equipped with a Phenomenex Kinetex reversed phase column (50 mm × 4.6 mm, 2.6 μm, C18, 100 Å); method: 10% (v/v) of acetonitrile (0.1% formic acid) in 90% (v/v) of H_2_O (0.1% formic acid), ramped to 100% acetonitrile (0.1% formic acid) over 5.5 min, and held at 100% acetonitrile (0.1% formic acid) for 1 min with a flow rate of 1.3 ml/min; UV detector, 254 nm. The purity of the compound was ≥95% in this analysis (Fig. S1). IR spectrum was recorded on a 26 Perkin-Elmer 65 IR spectrometer.

### 2.2 Plant material

The roots of *L. giberroa* Hemsl. were collected from Suba Menagesha Forest, located 45 km west of Addis Ababa in the Oromia region of Ethiopia. The plant was authenticated by Mr. Melaku Wondafrash, a senior botanist at the National Herbarium, College of Natural and Computational Sciences, AAU, where a voucher specimen was deposited (GT001).

### 2.3 Experimental animals and rodent parasites

Healthy, 5–6 weeks old Swiss albino mice of either sex weighing 24–28 g were used during the experiment. The mice were obtained from the Ethiopian Health and Nutritional Research Institute (EHNRI), Addis Ababa, Ethiopia. Mice were acclimatized for one week before the experiment. They were housed in an air-conditioned room, kept at room temperature, exposed to a 12 h light/dark cycle, allowed free access to standard pellets and water *ad libitum*.

Chloroquine sensitive *Plasmodium berghei* ANKA strain infected donor mice were obtained from Mekelle University, College of Health Sciences, Department of Pharmacology. The parasite was maintained by serial passage of blood from donor mice on a weekly basis. All procedures followed were in accordance with the Guidelines for the Care and Use of Laboratory Animals (NIH Guidelines for Care and Use of Laboratory Animals, 1996) and were approved by the Institutional Review Board of the School of Pharmacy (SoP), Addis Ababa University (AAU).

### 2.4 Extraction, fractionation and isolation

**Fig. 1:**
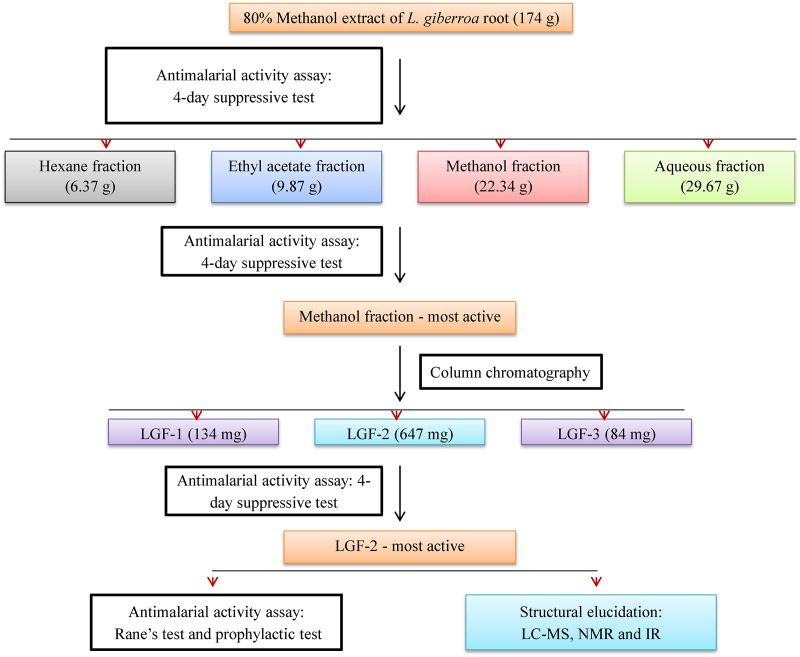
Bioassay-guided isolation of LGF-2 from the 80% methanol extract of the roots of *Lobelia giberroa*.

The powdered plant material (1000 g) was macerated with 80% methanol for 72 h with intermittent shaking (Fig. 1). The extract was filtered, and the mark was re-macerated two times with fresh solvent. The methanol was removed by using a rotary evaporator (BUCHI Rotavapor™ R-300, Switzerland), and the remaining water in the extract was removed by a lyophilizer (Alpha 1-2LD plus Martin Christ Co. Ltd., Osterode, Germany). The dried 80% methanol extract was then transferred into a vial and kept in a desiccator until use.

The dried 80% methanol extract was then successively extracted with solvents of differing polarities (hexane, ethyl acetate, methanol, and water) using a Soxhlet apparatus. The organic solvents were removed by a rotary evaporator, and the aqueous fraction was dried in a lyophilizer. The percent yields of dried hexane, ethyl acetate, methanol, and aqueous fractions were calculated and the fractions were stored in a desiccator until use.

Among the four fractions, the methanol fraction (with the highest antimalarial activity) was further fractionated by using silica gel packed (particle size of 0.2–0.5 mm) column chromatography. Gradient elution with 100% chloroform, followed by chloroform: methanol (9:1) and chloroform: methanol (8:2) afforded three column sub-fractions (LGF1-3). The purity of the most active sub-fraction (LGF-2) was confirmed by HPLC (Fig. S1) and analytical silicagel TLC.

### 2.5 Acute oral toxicity test

Acute oral toxicity test of the 80% methanol extract, solvent fractions and sub-fractions were conducted following the organization for economic co-operation and development (OECD) guideline 425 (OECD, 2008). Forty healthy (8 groups of five mice each), female albino mice of 6–8 weeks were used to test the acute oral toxicity of the 80% methanol extract, the 4 solvent fractions and the 3 sub-fractions. All mice were fasted for 4 h before and 2 h after administration of the test substances. First, a mouse from each group was given 2000 mg/kg of the test substance by using oral gavage. Since neither death nor gross changes such as loss of appetite, hair erection, lacrimation, tremors, convulsions, salivation, diarrhea, mortality, and other signs of toxicity were observed within 24 h, additional four mice were administered 2000 mg/kg of the test substance. Each animal was monitored for general symptoms of toxicity and/or mortality for 4 h at 30 min intervals and subsequently for 14 days at 24 h intervals.

### *2.6* *In vivo* antimalarial activity test

#### 2.6.1 Inoculation

A blood smear was prepared on microscope slides from blood films taken from the donor mouse tail. The smear was fixed with absolute methanol and stained with Giemsa to determine parasitemia level under a microscope (Primo Star, Carl Zeiss, Germany). When the donor parasitemia is approximately 30%, mice were sacrificed by cervical dislocation and blood was collected by cardiac puncture in a Petri dish with an anticoagulant, 0.5% trisodium citrate. The blood collected was then diluted with physiological saline (0.9%) in such a way that 1 ml blood contained 5 x 10^7^ infected erythrocytes. Each mouse intraperitoneally received 0.2 ml of diluted blood which contains 1 x 10^7^ *P. berghei* infected erythrocytes.

#### 2.6.2 Four-day suppressive test

Peter's four-day suppressive test was employed for the antimalarial evaluation of test substances. For the evaluation of the 80% methanol extract, twenty five inoculated mice were randomly divided into five groups each having five mice. Group I and II served as negative and positive controls, and 0.2 ml distilled water and 25 mg/kg/day chloroquine were administered to each group, respectively. The remaining groups (III, IV and V) were treated with the 80% methanol extract at 100, 200 and 400 mg/kg/day, respectively.

Similarly, for each fraction, twenty-five inoculated mice were randomly selected and divided into five groups, each having five mice. The mice in each group were randomly assigned into three treatment groups and two controls. For the hexane, ethyl acetate, and methanol fractions, their first group received 0.2 ml of the vehicle Tween 80 (2%) and for the aqueous fraction, its first group received 0.2 ml of distilled water. All the second groups were administered with 25 mg/kg/day standard chloroquine. The rest three treatment groups were given their respective fractions at a dose of 100, 200 and 400 mg/kg/day. Likewise, to evaluate the column sub-fractions, a similar procedure was followed, except, for the treatment groups receiving their respective sub-fractions at a dose of 25, 50, and 100 mg/kg/day.

All the test substances were administered orally using oral gavage. Treatment was started 2 h post-infection on day 0 and continued daily for 3 days (i.e., from day 0 to day 3). On the fifth day (or day 4), Giemsa-stained thin blood film was prepared from the tail of each mouse to count the number of parasites under the microscope with an oil immersion objective of 100 x magnification power. The parasitemia was determined by counting a minimum of five fields per slide (Peters, 1965; Hilou et al., 2006). Percent parasitemia and percent parasitemia inhibition was calculated using the formula:

**Figure:**
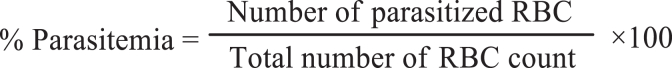

**Figure:**
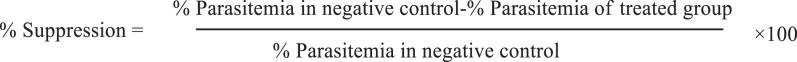

#### 2.6.3 Body weight and survival time measurement

Body weight and mean survival time parameters were used to evaluate the *in vivo* antimalarial activity of the test substances. The body weight of each mouse was determined on day 0 before infection and day 4 using a sensitive digital weighing balance. Survival time was recorded from day 1 to day 28 post inoculation. Then, the mean body weight and mean survival time were calculated for each group (Mesfin et al., 2012).

**Figure:**
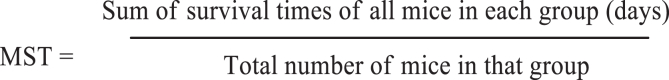

#### 2.6.4 Packed cell volume and rectal temperature measurement

Packed cell volume (PCV) and rectal temperature parameters were also used to assess the *in vivo* antimalarial activity of the test substances. To measure PCV, blood was collected from the tail of each mouse in heparinized micro hematocrit capillary tube to 3/4th its height and sealed at their dry end with a sealing clay. The capillary tubes were centrifuged at 12,000 rpm for 5 min in a microhematocrit centrifuge. Rectal temperature was measured using a digital rectal thermometer. The mean rectal temperature and PCV were then calculated (Fentahun et al., 2017).

PCV was determined using the following formula by.

**Figure:**
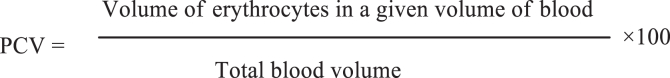

#### 2.6.5 Curative activity test (Rane's test)

The curative potential of the isolated compound (LGF-2) was evaluated following the method described by Ryley and Peters (1970). In the test, twenty five inoculated mice were randomly assigned into five groups each having five mice on the 4th day (72 h post-inoculation). Group I and II served as negative and positive controls each administered with 0.2 ml of distilled water and of 25 mg/kg/day standard pure chloroquine, respectively. The remaining treatment groups were given 25, 50, and 100 mg/kg/day doses of the compound (LGF-2). Treatments were continued for the next 3 days (i.e., 96, 120, 144 h post-inoculation). Geimsa stained thin blood film was prepared using blood from the tail of each mouse daily for 5 days to monitor parasitemia levels. Body weight, PCV and rectal temperature of the mice were also recorded on the 4th (before treatment) and 8th days (24 h after the last treatment). The mice were followed for 28 days post-inoculation. Survival time was recorded from day 1 to day 28 post-inoculation and the mean survival time was calculated for each group (Nardos and Makonnen, 2017).

#### 2.6.6 Prophylactic activity test (Peter's repository test)

The prophylactic potential of the compound (LGF-2) was evaluated as described by Peters (1965). Accordingly, twenty five mice were randomly distributed into 5 groups each having five mice. Group I and II served as negative and positive controls each administered with 0.2 ml of distilled water and 25 mg/kg/day of standard pure chloroquine, respectively on the 1st day. The rest of the groups were similarly treated on the 1st day with the compound (LGF-2) at a dose of 25, 50, and 100 mg/kg/day. Treatments continued for the next three days. On the 5th day, all the mice were inoculated with 0.2 ml blood containing 1 × 10^7^ *P. berghei* infected erythrocytes. On the 8th day, blood smears were prepared from each mouse and the parasite level was determined. Body weight, PCV, and temperature were recorded on the 5th day before inoculation. The mice were followed for 28 days post-inoculation. Survival time was recorded, and the mean survival time was calculated for each group.

### 2.7 Data analysis

The data were entered into Statistical Package for Social Sciences (SPSS) version 25. The results were summarized as mean ± standard error of the mean (SEM). One-way analysis of variance (ANOVA) followed by Tukey's post hoc test was used to compare differences in mean among the groups. A *P* value of less than 0.05 was considered statistically significant.

## 3 Results and discussion

### 3.1 Acute oral toxicity

Long-term use of a medicinal plant without any evidence of risk may imply that the medicinal plant is harmless. However, the absence of any documented side-effects does not guarantee that herbal treatments are safe. Thus, to provide criteria for defining a safe dose for human use, a detailed toxicological evaluation must be undertaken using appropriate experimental animals.

Despite its widespread use in traditional medicine, there are no reports of oral toxicity investigations on *L. giberroa*. Thus, the acute oral toxicity of the 80% methanol extract and solvent fractions of the roots of *L. giberroa,* as well as lobetyolin (LGF-2) isolated from the methanol fraction of *L. giberroa* roots*,* was evaluated. In the current study, there were no treatment-related deaths or toxic symptoms, indicating that the extract, solvent fractions, and lobetyolin have an LD_50_ of larger than 2000 mg/kg in mice.

### 3.2 Antimalarial activity of the 80% methanol extract

**Fig. 2:**
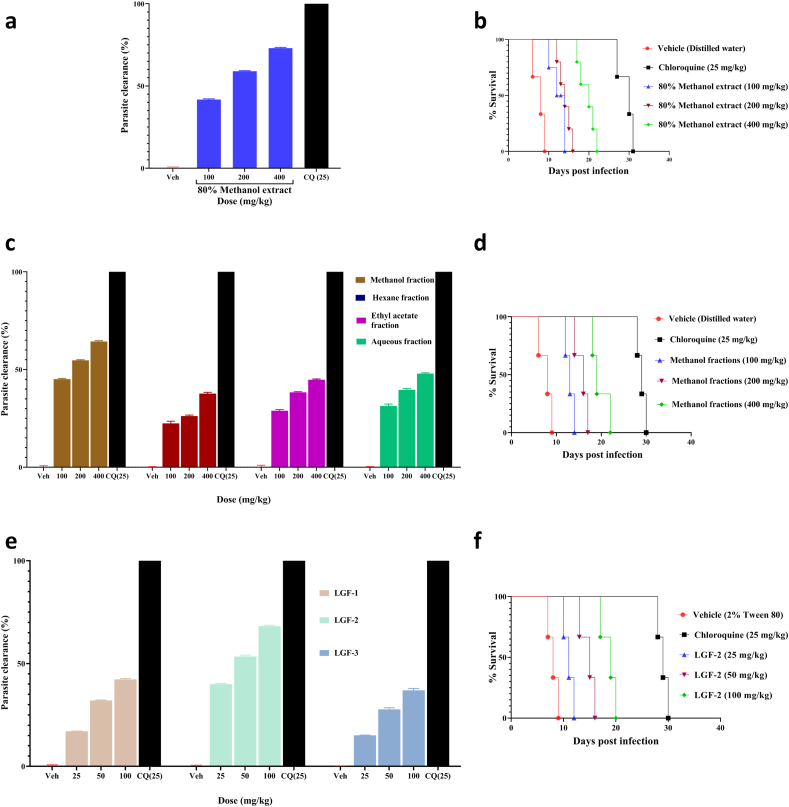
Antimalarial activity of the 80% methanol extract and fractions of the roots of *Lobelia giberroa* against mice infected with *Plasmodium berghei* in the 4-day suppressive test. % clearance on day 4 post-infection is calculated and survival curves of each mouse are plotted as percent survival/group for the evaluation of suppression of early stage infection. (A) % clearance of the 80% methanol extract. (B) Survival plots of each mouse administered with the 80% methanol extract (100, 200 and 400 mg/kg), chloroquine (CQ), and distilled water. (C) % clearance of the solvent fractions. (D) Survival plots of each mouse administered with the methanol fraction (100, 200 and 400 mg/kg), chloroquine (CQ), and distilled water (E) % clearance of the column sub-fractions. (F) Survival plots of each mouse administered with LGF-2 (25, 50 and 100 mg/kg), chloroquine (CQ), and distilled water. Values are presented as mean ± SEM; n = 5.

In the four-day suppressive test, the 80% methanol extract of the roots of *L. giberroa* showed significant (*p* < 0.001) parasite suppression in a dose-dependent manner with a chemosuppression value of 41.72, 58.95, and 73.05% at doses of 100, 200, and 400 mg/kg, respectively (Fig. 2a). Additionally, the extract at all the doses tested significantly prolonged the mean survival time of treated mice (Fig. 2b).

Anemia, body weight loss and body temperature reduction are the general features of malaria infected mice (Lamikanra et al., 2007). *P*. *berghei* causes reduction in rectal temperature of mice due to reduction in metabolic rates that occur because of increased parasitemia. Therefore, antimalarial agents are expected to prevent anemia, thwart body weight loss and stabilize body temperature in infected mice due to the rise in parasitemia level. The 80% methanol extract of *L. giberroa* significantly prevented weight loss and rectal temperature and PCV reduction associated with an increase in parasitemia level (Table 1). Nevertheless, the 80% methanol extract exhibited lower activity (*p* < 0.001) compared to the standard drug chloroquine in terms of all test parameters.

**Table 1:** Packed cell volume, rectal temperature and body weight of *Plasmodium berghei* infected mice before and after administration of the 80% methanol extract of the roots of *Lobelia giberroa* in the four-day suppressive test.

Group	Dose (mg/kg)	Weight			Temperature			Packed cell volume		
		D0	D4	% Change	D0	D4	% Change	D0	D4	% Change
DW		26.92 ± 0.29	21.18 ± 0.30	−21.3 ± 1.16	36.9 ± 0.09	33.72 ± 0.28	−8.61 ± 0.9	56.3 ± 0.62	47.18 ± 0.69	−16.18 ± 1.11
CQ	25	26.7 ± 0.45	26.86 ± 0.42	0.61 ± 0.16^a3^	36.96 ± 0.13	36.58 ± 0.23	−1.03 ± 0.37^a3^	55.42 ± 0.92	54.00 ± 1.09	−2.58 ± 0.59^a3^
LGCE	100	26.76 ± 0.59	22.42 ± 0.77	−16.24 ± 1.86^b3d1e3^	36.82 ± 0.19	34.78 ± 0.17	−5.5 ± 0.58^a2b3d2e3^	54.64 ± 0.88	48.00 ± 0.63	−12.12 ± 0.86^a1b3e2^
	200	25.14 ± 0.76	22.72 ± 0.66	−9.47 ± 2.44^a3b2e1^	36.84 ± 0.05	36.04 ± 0.12	−2.17 ± 0.36^a3^	54.48 ± 0.82	48.16 ± 1.03	−11.63 ± 0.61^a2b3e2^
	400	26.26 ± 0.56	24.57 ± 0.52	−6.44 ± 0.64^a3^	36.94 ± 0.09	36.64 ± 0.09	−0.81 ± 0.12^a3^	54.66 ± 0.96	50.08 ± 1.09	−7.08 ± 0.71^a3^

Data are expressed as mean ± SEM; n = 5; compared to a, negative control; b, CQ25 mg/kg; c, 100 mg/kg; d, 200 mg/kg; e, 400 mg/kg; 1, p < 0.05; 2, p < 0.01; 3, p < 0.001; DW, distilled water; CQ, chloroquine; D0, pre-treatment value on day 0; D4, post-treatment value on day 4; LGCE, 80% methanol extract of the roots of *Lobelia giberroa.*

Because the 80% methanol extract showed greater than 50% suppression at 200 mg/kg, it is considered as a potential schizontocidal agent in early erythrocytic infection (Deharo et al., 2001). More importantly, the 19 days mean survival time of mice treated with 400 mg/kg of the 80% methanol extract is a strong indicator of the ability of the extract to reduce the overall pathogenic effect of the parasite. Nevertheless, the extract did not cure the infection, as the mean survival time of treated mice was less than 27 days (Slack et al., 2012). This might be attributed to the recrudescence of the parasites after significantly extended suppression. To the best of our knowledge, this is the first report describing the antimalarial activity of the genus *Lobelia*, and thus the plant.

### 3.3 Antimalarial activity of the solvent fractions

Among the solvent fractions, the methanol fraction showed the highest antimalarial activity in the four-day suppressive test with a chemosuppression of 64.37% at a dose of 400 mg/kg, followed by the aqueous (47.92%), ethyl acetate (44.80%), and hexane (37.21%) fractions (Fig. 2c). Also, the survival time of the methanol fraction treated mice was longer than the mice treated with the other fractions (Fig. 2d). Compared to the 80% methanol extract, the methanol fraction had lesser chemo-suppressive effect. All the solvent fractions significantly (*P* < 0*.*001) suppressed infection-induced reduction of body weight, rectal temperature, and PCV. The positive control, chloroquine, showed a curative chemosuppression and a marked stabilization of body weight, rectal temperature, and PCV (Table 2; Table S1).

**Table 2:** Body weight, rectal temperature and packed cell volume of *Plasmodium berghei* infected mice before and after administration of the methanol fraction of the roots of *Lobelia giberroa* in the four-day suppressive test.

Group	Dose (mg/kg)	Weight			Temperature				Packed cell volume		
		D0	D4	%Change	D0	D4	%Change		D0	D4	%Change
TW80		26.52 ± 0.39	21.82 ± 0.42	−17.72 ± 1.09	37.08 ± 0.73	33.42 ± 0.92	−9.87 ± 0.23		56.18 ± 0.82	49.92 ± 0.70	−11.13 ± 0.49
CQ	25	26.92 ± 0.64	27.30 ± 0.69	1.41 ± 0.42^a3^	37.14 ± 0.07	36.90 ± 0.07	−0.64 ± 0.06^a3^		55.40 ± 0.93	55.08 ± 0.92	−0.58 ± 0.13^a3^
MF	100	26.68 ± 0.65	22.80 ± 0.58	−14.54 ± 0.38b3e3d1	36.80 ± 0.08	34.31 ± 0.16	−7.52 ± 0.78a3b3d2e3		55.50 ± 1.07	51.36 ± 1.40	−7.52 ± 0.78a3b3e3
	200	26.40 ± 0.78	23.60 ± 0.80	−10.63 ± 0.89a3b3e3	37.04 ± 0.08	35.36 ± 0.19	−5.59 ± 0.15a3b3e3	55.35 ± 0.79		52.26 ± 0.83	−5.59 ± 0.15a3b3e1
	400	27.28 ± 0.71	25.94 ± 0.98	−4.91 ± 0.99^a3b3^	36.88 ± 0.12	36.26 ± 0.28	−3.16 ± 0.54^a3^		54.48 ± 1.15	52.76 ± 1.14	−3.16 ± 0.54^a3b1^

Data are expressed as mean ± SEM; n = 5; a, compared to negative control; b, to CQ25 mg/kg; c, to 100 mg/kg; d, to 200 mg/kg; e, to 400 mg/kg; 1, p < 0.05; 2, p < 0.01; 3, p < 0.001; TW80, 2% Tween80; CQ, chloroquine; MF, methanol fraction; D0, pre-treatment value on day 0; D4, post-treatment value on day 4.

The methanol fraction is considered to have good antimalarial activity, because it suppresses malaria parasites by more than 50% at 200 mg/kg (Deharo et al., 2001). The prolonged survival days and prevention of body weight loss and rectal temperature drop suggest the fraction's action on the overall pathology of the disease and its strong parasite clearance. Glycosides and other polar components that are supposedly found in the methanol fraction could be responsible for the more pronounced effect (Park et al., 2008).

### 3.4 Antimalarial activity of the column sub-fractions

Column fractionations of the most active solvent (methanol) fraction led to the collection of three column sub-fractions: LGF-1, LGF-2, and LGF-3, and LGF-2 was found to be a pure compound (Fig. S1). All the column sub-fractions displayed a significant dose-dependent antimalarial activity in the four-day suppressive test (Fig. 2e). Moreover, the pure compound (LGF-2) showed the highest parasite chemosuppression, 68.21% at 100 mg/kg. It also resulted in a longer mean survival time in mice, 18 days at 100 mg/kg (Fig. 2f). All the sub-fractions produced a significant effect on the reduction of body weight loss, PCV and rectal temperature drop. However, chloroquine treated groups had a more pronounced effect than the sub-fractions treated groups (Table 3; Table S2).

**Table 3:** Body weight, rectal temperature and packed cell volume of *Plasmodium berghei* infected mice before and after administration of the compound (LGF-2) in the four-day suppressive test.

Group	Dose mg/kg	Weight			Temperature			Packed cell volume		
		D0	D4	%Change	D0	D4	%Change	D0	D4	%Change
TW80		25.86 ± 0.49	21.50 ± 0.44	−16.86 ± 0.71	36.70 ± 0.19	32.84 ± 0.17	−10.51 ± 0.40	54.76 ± 0.41	46.88 ± 0.50	−14.38 ± 0.96
CQ	25	25.60 ± 0.52	26.36 ± 0.59	2.95 ± 0.50^a3^	36.72 ± 0.09	36.54 ± 0.09	−0.49 ± 0.13^a3^	54.04 ± 0.79	53.70 ± 0.82	−0.6321 ± 0.12^a3^
LGF-2	25	26.14 ± 0.99	23.12 ± 0.97	−11.61 ± 0.39^a3b3d3e3^	37.02 ± 0.14	34.00 ± 0.14	−8.16 ± 0.15^a3b3e3^	54.20 ± 0.98	48.32 ± 0.84	−10.84 ± 0.19^a2b3d1e3^
	50	24.88 ± 0.79	23.10 ± 0.65	−7.11 ± 0.59^a3b3e3^	36.72 ± 0.10	34.20 ± 0.20	−6.86 ± 0.41^a3b3e3^	54.98 ± 0.83	50.57 ± 0.79	−8.02 ± 0.32^a3b3e3^
	100	26.90 ± 0.35	26.14 ± 0.39	−2.83 ± 0.61^a3b3^	36.98 ± 0.11	35.64 ± 0.07	−3.62 ± 0.33^a3b3^	54.10 ± 0.60	51.84 ± 0.79	−4.19 ± 0.59^a3b3^

Data are expressed as mean ± SEM; n = 5; a, compared to negative control; b, to CQ25 mg/kg; c, to 25 mg/kg; d, to 50 mg/kg; e, to 100 mg/kg; 1, p < 0.05; 2, p < 0.01; 3, p < 0.001; TW80, 2% Tween80; CQ, chloroquine; D0, pre-treatment value on day 0; D4, post-treatment value on day 4.

The compound (LGF-2) showed greater than 50% parasitemia suppression at 50 mg/kg. Thus, it can be considered as a promising antimalarial agent (Soares et al., 2015). Besides, the 18 days mean survival time of 100 mg/kg LGF-2 treated mice indicates that the compound has the ability to alter the overall pathology of malaria in the mice. On the other hand, even at 100 mg/kg, LGF-2 was unable to completely clear parasites; hence, the parasites eventually returned. The effect of LGF-2 on body weight and temperature suggest that it might have mild antifebrile effect (Ang et al., 2000; Alebachew et al., 2021).

### 3.5 Structural elucidation

Bioassay-guided fractionation of the 80% methanol extract of the roots of *L. giberroa* resulted in the isolation of an active compound (LGF-2), Rf = 0.56 (Chloroform/Methanol; 4:1). The compound (LGF-2) was isolated as a yellow solid. The ^13^C NMR spectrum of LGF-2 (Fig. S2) revealed a total of twenty carbons. The signals at δ 28.94, δ 32.55, δ 60.58 and δ 61.55 indicated the presence of four CH_2_ groups as confirmed by the negative signal on DEPT-135 (Fig. S3). From the 12 positive signals found on DEPT-135 spectrum, only 11 were observed in DEPT-90 spectrum (Fig. S4). This showed that the presence of 11 CH groups at δ 65.08, δ 70.61, 77.37, δ 77.44, δ 73.75δ, δ 80.02, δ 100.54, δ 109.66, δ 125.53, δ 136.62 and δ 145.48; the signal at δ 19.12 was confirmed the presence of one CH_3_ group. The downfield CH_2_ signals at δ 60.58 and δ 61.55 indicate that they are oxygenated methylene groups. The signals at δ 69.57, δ 72.46, δ 77.55 and δ 83.31, which were observed neither in the DEPT-135 nor in the DEPT-90, were therefore four quaternary carbons. The downfield ^13^C NMR signals at δ 109.64, δ 145.47 and δ 125.51, δ 136.61 supported the presence of two double bonds. In addition, the signal at δ 100.54 was typical of anomeric carbon and indicated the presence of sugar moiety in the structure. Furthermore, the peaks at δ 61.53, δ 69.55, δ 73.73, δ 77.43 and δ 77.53 signified the five sugar carbons. Table 4 summarizes the ^13^C NMR data of the compound (LGF-2).

**Table 4:** ^1^H and^13^C NMR data of compound (LGF-2) measured in DMSO-*d*_6_.

Present data				Reference data (Ishimaru et al., 1991)	
Position	δ_C_ (ppm)		δ_H_ (ppm)	δ_C_ (ppm)	δ_H_ (ppm)
1	19.12	1.80 (3H, dd, *J* = 1.9, 6.9 Hz)		19.20	1.73 (3H, dd, J = 2.3, 6.8, Hz)
2	145.48	6.39 (1H, dq, J = 6.8, 16.1, Hz)		144.70	6.29 (1H, dq, J = 6.8, 16.2,Hz)
3	109.66	5.71 (1H, dd, J = 2.1, 15.9, Hz)		110.40	5.55 (1H, *dd*, *J* = 2.3, 16.2, Hz)
4	83.31	–		82.40	–
5	77.55	–		78.20	–
6	72.46	–		72.90	–
7	69.57	–		70.80	–
8	65.08	4.45 (1H, d, J = 5.4, Hz)		65.60	4.40 (1H, *d*, *J* = 6.8, Hz)
9	80.02	4.04 (1H, t, J = 7.1, Hz)		81.80	4.20 (1H, *t*, *J* = 6.8, Hz)
10	125.53	5.37 (1H, dd, J = 7.4, 15.5, Hz)		126.40	5.4 (1H, dd, J = 6.8, 16.2, Hz)
11	136.62	5.82 (1H, dt, J = 6.8, 14.5, Hz)		136.70	5.86 (1H, *dt*, *J* = 6.8, 16.2, Hz)
12	28.94	2.07 (2H, q, J = 7.2, Hz)		28.90	2.09 (2H*, br*, *dd*, *J* = 6.8, 13.5 Hz)
13	32.55	1.50 (2H, q, J = 6.6, Hz)		33.00	1.56 (2H, *q*, *J* = 6.8, Hz)
14	61.55	3.41 (2H, t, J = 6.9, Hz)		61.90	3.49 (2H*, t*, *J* = 6.8, Hz)
1′	100.54	4.15 (1H, d, J = 7.7, Hz)		100.80	4.32 (lH, d, J = 7.5, Hz)
2′	73.75	3.0 (1H m)		74.70	3.24 (1H, *m*)
3′	77.44	3.0 (1H, m)		77.90	3.24 (1H, *m*)
4′	70.61	3.10 (1H, t, *J* = 8.3 Hz)		71.50	3.34 (1H, *t*, *J* = 8.3, Hz)
5′	77.37	3.0 (1H, m)		77.80	3.24 (1H, m)
6′	60.58	3.65 (1H, d, J = 11.7, Hz)		62.00	3.77 (1H*, dd*, *J* = 6.8, 12.1, Hz)

*s* = singlet, *d* = doublet, *dd* = doublet of doublets, *m* = multiplet, *br* = broad, *q* = quartet, *t* = triplet.

In the ^1^H NMR spectrum (Fig. S5), the proton signal at δ 4.15 (d, *J* = 7.7 Hz, 1H) further indicated the presence of an anomeric proton, thereby the presence of a sugar unit. The signals at δ 1.50, δ 2.07 and δ 3.41 each were integrated to two protons that were attributed to be CH_2_ protons. The downfield shifted CH_2_ signal (δ 3.41) was an oxygenated methylene and the proton signal at δ 1.80 (3H, *dd*, *J* = 1.9, 6.9 Hz, H-1) strengthened the presence of a methyl group which was found in proximity to olefinic carbons. The proton signals at δ 4.45 (1H, *d*, *J* = 5.4 Hz, H-8) and δ 4.04 (1H, *t*, *J* = 7.1 Hz, H-9) were integrated to one proton showed the presence of two CH protons. The two pairs of trans coupled peaks δ 5.37 (1H, *dd*, *J* = 7.4, 15.5 Hz, H-10), δ 5.82 (1H, *dt*, *J* = 6.8, 14.5 Hz, H-11) and δ 6.39 (1H, *dq*, *J* = 6.8, 16.1 Hz, H-2), δ 5.71 (1H, *dd*, *J* = 2.1, 15.9 Hz, H-3) protons supported the presence of two double bond carbons. These double bonds were assigned as *E* due to the large vicinal coupling constant (*J* = 15.5 Hz and 14.5 Hz) between alkene protons at C-10 and C-11, and between alkene protons at C-2 and C-3 (*J* = 16.1 Hz and 15.9 Hz) (Ishimaru et al., 1991; Dumlu et al., 2008). Table 4 summarizes the ^1^H NMR data of the compound (LGF-2).

The HMBC spectrum of LGF-2 (Fig. S6) showed the connection of CH_3_ protons at C-1 to the olefinic carbons at C-2 and C-3. The olefinic proton at C-11 correlated with two CH_2_ carbons at C-12, C-13, and CH carbon at C- 9. The C-12 of LGF-2 showed a long-range correlation with the proton located at olefinic carbon at C-10. The two protons on the oxygenated CH_2_ group at C-14 displayed correlations with carbons at C-12 and C-13. This long-range correlation has also been displayed by the CH proton at C- 8 and C- 9. The large coupling constant (*J* = 7.7 Hz) of H-l’ revealed the β-configuration of these anomeric centers (Ishimaru et al., 1991). Furthermore, the ^1^H–^1^H COSY spectrum of LGF-2 (Fig. S7) exhibited correlation cross-peaks between CH_3_ (C-l) protons and CH (C-2) proton, CH (C-2) proton and CH (C-3) proton, CH (C-8) proton CH (C-9) and CH (C-l0) proton, CH (C-l0) proton and CH (C-11) proton, CH (C-11) proton and CH_2_ (C-12) protons, CH_2_ (C-12) protons and CH_2_ (C-13) protons, and CH_2_ (C-13) protons and CH_2_OH (C-14) protons.

The mass spectrum of LGF-2 (Fig. S8) gave a pseudomolecular ion peak at *m/z* 414.1 [M + NH_4_^+^] ^+^ (Calc. m/z 414.46998[M + NH_4_^+^] ^+^). The base peak at 217 indicated the presence of sugar moiety, pyranosides. The signal at 199 represented [(M/2) +H^+^] fragment peaks. In addition, the fragment peaks at *m/z* 361 and 379 were [M-2OH] and [M – OH], respectively indicating LGF-2 is an alcohol. The molecular ion peak of alcohols is small and sometimes undetectable due to incomplete conversion and unexpected reactions (Traeger and Morton, 2004; Sayed et al., 2010). As a result, the molecular ion peak of the LGF-2 was absent. The molecular weight of LGF-2 was deduced to be 396, and its molecular formula was determined to be C_20_H_28_O_8._

The IR spectrum of LGF-2 (Fig. S9) signaled the presence of O–H, C–O, C–H stretch of CH_3,_ C–H stretch of CH_2_, and CC functional groups stretch. The broad bands at 3379 cm^−1^ and 1070 cm^−1^ showed the hydroxyl and C–O stretches respectively. The bands at 2922 cm^−1^ and 2854^−cm^ gave evidence for the existence of sp3 hybridized C–H stretch and the weak band at 2229 cm^−1^ indicated acetylenic carbon stretch. The band at 1619 showed the presence of alkene. Therefore, the IR suggested that LGF-2 contains triple bond, alcohol, alkenes and C–O.

**Fig. 3:**
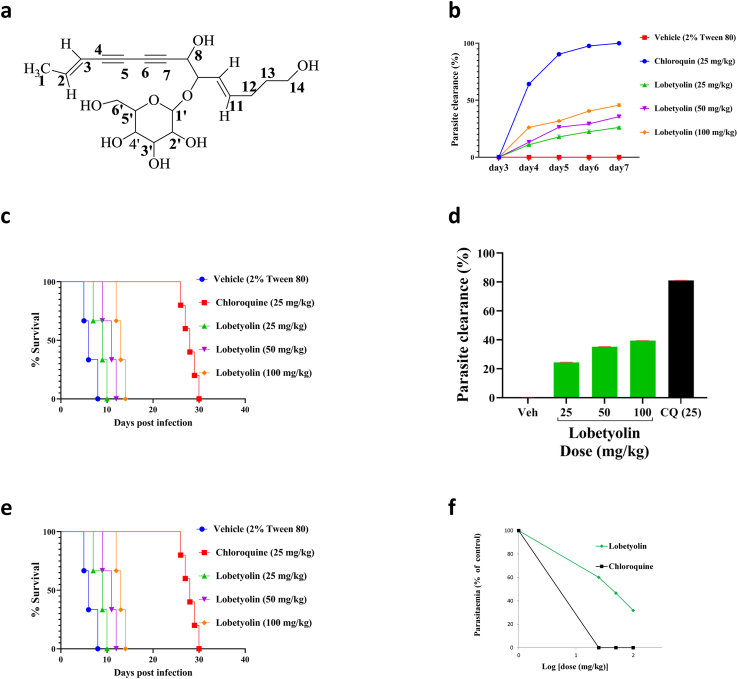
Chemical structure and antimalarial activity of lobetyolin. (A) Chemical structure of lobetyolin (LGF-2). (B) % clearance of lobetyolin on day 3 to day 7 post-infection is calculated and (C) survival curves of each mouse are plotted as percent survival/group for the evaluation of suppression of established infection in the Rane's test. (D) % clearance of lobetyolin on day 8 post-treatment is calculated and (E) survival curves of each mouse are plotted as percent survival/group for the evaluation of prevention of infection in the prophylactic test. (F) The ED_50_ of lobetyolin is estimated from a plot of log dose against parasitaemia (expressed as a percentage of the control) in the 4-day suppressive test. Values are presented as mean ± SEM; n = 5.

Based on the above results and in comparison with the reported ^1^H NMR and ^13^C NMR data (Ishimaru et al., 1991, 2003; Dumlu et al., 2008), LGF-2 was identified as lobetyolin (**1**) (Fig. 3a). Lobetyolin was isolated from the roots of *L. giberroa* for the first time.

### 3.6 Curative and prophylactic antimalarial activity of lobetyolin

#### 3.6.1 Curative activity

Lobetyolin displayed a dose-dependent suppressive effect against established *P. berghei* infection in mice in Rane's curative model (Fig. 3b). The compound significantly prolonged the mean survival days of the treated groups. However, compared to the standard drug, it was less effective (Fig. 3c). At all doses, lobetyolin suppressed body weight loss, and rectal temperature, and PCV reduction (Table S3).

#### 3.6.2 Prophylactic activity

Lobetyolin produced significant (p < 0.001) prophylactic parasitemia suppression against *P. berghei* infection in mice (Fig. 3d). The compound extended survival time of treated mice (Fig. 3e), and significantly attenuated body weight loss and rectal temperature and PCV reduction (Table S4).

The ED_50_ of lobetyolin was estimated to be 36.8 mg/kg from the dose-response plot of the four-day suppression test (Fig. 3f). This entails that the compound has a more potent effect in the early erythrocytic stage of parasite infection than the 80% methanol extract and fractions. However, the ED_50_ of lobetyolin was lower when compared with the antimalarial drugs chloroquine, artemisinin derivatives, and some other compounds, which had ED_50_ in the range of 0.4–18 mg/kg, (Brossi et al., 1988; Ancelin et al., 2003; Chu et al., 2019). This could be attributed to lobetyolin's partial water solubility, which results in limited bioavailability (Dong et al., 2021). This is further supported by the recrudescence noted in the four-day suppression test after 73% suppression. Thus, structural optimization of lobetyolin for example by esterification of the OH groups with amine containing polar substituents such as lysine to enhance its water solubility (bioavailability) is recommended.

Preclinical studies revealed that lobetyolin, a C_14_-polyacetylene glucoside, possess anticancer (He et al., 2019; Bailly, 2021; Chen et al., 2021), antioxidant (Dumlu et al., 2008), and antigout properties (Yoon and Cho, 2021). In spite of the availability of more than five hundred polyacetylenes natural products, only eight were tested for their antimalarial activity (Negri, 2015; Folquitto et al., 2019). Moreover, lobetyolin, in the present study, (R)-1, 2-dihydroxy-trideca-3,5,7,9,11-pentayne (DTP) isolated from *Bidens pilosa* (Tobinaga et al., 2009), and acetylenic thiophenes from *Echinops hoehnelii* (Bitew et al., 2017) are the only ones evaluated *in vivo*. DTP is a fairly unstable C_13_-polyacetylene (aglycon) having potent *in vivo* antimalarial activity with a chemosuppression value of 63.1% at 0.8 mg/kg dose after an IV administration (Tobinaga et al., 2009). Interestingly, the instability and potent antimalarial property of DTP offers three crucial insights regarding lobetyolin. First, the glucose moiety of lobetyolin is important for its stability. Notably, lobetyolin was found unstable upon hydrolysis. Second, as mentioned above and indicated by Dong et al. (2021), the poor bioavailability of lobetyolin after an oral route undermines its curative potency or complete parasite clearance. Third, the *in vivo* potency of DTP (after IV route) and lobetyolin (after oral route) again cements the presence of a specific target for the compounds in the parasite. However, because of the absence of prior investigation on the mechanism of action of polyacetylenes and/or related compounds, it is difficult to suggest plausible target/s for lobetyolin. Thus, target deconvolution is highly desirable. To the best of our knowledge, this is the first report on the antimalarial activity of lobetyolin.

## 4 Conclusion

The results of this phenotypic bioassay-guided isolation of lobetyolin support the traditional claim of the roots of *L. giberroa*. Lobetyolin, the principal active compound of the plant, has the potential to serve as an early lead compound for the development of new antimalarial drugs. Exploration of structure-activity relationships through semi-synthetic modifications and combination studies with other antimalarial drugs are suggested as potential strategies to improve the antimalarial activity of lobetyolin.

## Declaration of competing interest

The authors declare no conflict of interest.

## References

[bib1] Abate G., Demissew S. (1989). Etse debdabe: Ethiopian traditional medicine.

[bib2] Alebachew Y., Bisrat D., Tadesse S., Asres K. (2021). *In vivo* anti-malarial activity of the hydroalcoholic extract of rhizomes of *Kniphofia foliosa* and its constituents. Malar. J..

[bib3] Ancelin M.L., Calas M., Bonhoure A., Herbute S., Vial H.J. (2003). *In vivo* antimalarial activities of mono-and bis quaternary ammonium salts interfering with *Plasmodium* phospholipid metabolism. Antimicrob. Agents Chemother..

[bib4] Ang K.K., Holmes M.J., Higa T., Hamann M.T., Kara U.A. (2000). *In vivo* antimalarial activity of the beta-carboline alkaloid manzamine A. Antimicrob. Agents Chemother..

[bib5] Bailly C. (2021). Anticancer properties of lobetyolin, an essential component of *Radix codonopsis* (Dangshen). Nat. Prod. Bioprospect..

[bib6] Bitew H., Mammo W., Hymete A., Yeshak M.Y. (2017). Antimalarial activity of acetylenic thiophenes from *Echinops hoehnelii* Schweinf. Molecules.

[bib7] Brossi A., Venugopalan B., Dominguez Gerpe L., Yeh H.J.C., Flippen-Anderson J.L., Buchs P., Luo X.D., Milhous W., Peters W. (1988). Arteether, a new antimalarial drug: synthesis and antimalarial properties. J. Med. Chem..

[bib8] Chekole G. (2017). Ethnobotanical study of medicinal plants used against human ailments in Gubalafto District, Northern Ethiopia. J. Ethnobiol. Ethnomed..

[bib9] Chen Y., Tian Y., Jin G., Cui Z., Guo W., Zhang X., Liu X. (2021). Lobetyolin inhibits the proliferation of breast cancer cells via ASCT2 down-regulation-induced apoptosis. Hum. Exp. Toxicol..

[bib10] Chu X.M., Wang C., Wang W.L., Liang L.L., Liu W., Gong K.K., Sun K.L. (2019). Triazole derivatives and their antiplasmodial and antimalarial activities. Eur. J. Med. Chem..

[bib11] Deharo E., Bourdy G., Quenevo C., Munoz V., Ruiz G., Sauvain M. (2001). A search for natural bioactive compounds in Bolivia through a multidisciplinary approach. Part V. Evaluation of the antimalarial activity of plants used by the Tacana Indians. J. Ethnopharmacol..

[bib12] Dong J., Cheng M., Xue R., Deng C., Liu H., Zhang T., Lu T., Mao C., Xiao S., Li L., Pi W. (2021). Comparative pharmacokinetic and bioavailability study of lobetyolin in rats after administration of lobetyolin and *Codonopsis pilosula* extract by ultra-performance LC–tandem mass spectrometry. Biomed. Chromatogr..

[bib13] Dumlu M.U., Gurkan E., Tuzlaci E. (2008). Chemical composition and antioxidant activity of *Campanula alliariifolia*. Nat. Prod. Res..

[bib14] Fentahun S., Makonnen E., Awas T., Giday M. (2017). *In vivo* antimalarial activity of crude extracts and solvent fractions of leaves of *Strychnos mitis* in *Plasmodium berghei* infected mice. BMC Complement. Altern. Med.

[bib15] Folquitto D.G., Swiech J.N., Pereira C.B., Bobek V.B., Halila Possagno G.C., Farago P.V., Miguel M.D., Duarte J.L., Miguel O.G. (2019). Biological activity, phytochemistry and traditional uses of genus *Lobelia* (Campanulaceae): a systematic review. Fitoterapia.

[bib16] Girum T., Shumbej T., Shewangizaw M. (2019). Burden of malaria in Ethiopia, 2000-2016: findings from the global health estimates 2016. Trop. Dis. Travel med. Vaccines.

[bib18] Hilou A., Nacoulma O.G., Guiguemde T.R. (2006). *In vivo* antimalarial activities of extracts from *Amaranthus spinosus* L. and *Boerhaavia erecta* L. in mice. J. Ethnopharmacol..

[bib19] Ishimaru K., Osabe M., Yan L., Fujioka T., Mihashi K., Tanaka N. (2003). Polyacetylene glycosides from *Pratia nummularia* cultures. Phytochemistry.

[bib20] Ishimaru K., Yonemitsu H., Shimomura K. (1991). Lobetyolin and lobetyol from hairy root culture of *Lobelia inflata*. Phytochemistry.

[bib21] Lamikanra A.A., Brown D., Potocnik A., Casals-Pascual C., Langhorne J., Roberts D.J. (2007). Malaria anemia of mice and men. Blood.110.

[bib22] Mennai I., Sifaoui I., Esseid C., López-Arencibia A., Reyes-Batlle M., Benayache F., Benayache S., Bazzocchi I.L., Lorenzo-Morales J., Piñero J.E., Jiménez I.A. (2021). Bio-guided isolation of leishmanicidal and trypanocidal constituents from *Pituranthos battandieri* aerial parts. Parasitol. Int..

[bib23] Mesfin A., Giday M., Animut A., Teklehaymanot T. (2012). Ethnobotanical study of antimalarial plants in Shinile District, Somali Region, Ethiopia, and *in vivo* evaluation of selected ones against *Plasmodium berghei*. J. Ethnopharmacol..

[bib24] Nardos A., Makonnen E. (2017). *In vivo* antiplasmodial activity and toxicological assessment of hydroethanolic crude extract of *Ajuga remota*. Malar. J..

[bib25] Negri R. (2015). Polyacetylenes from terrestrial plants and fungi: recent phytochemical and biological advances. Fitoterapia.

[bib26] NIH Guidelines for Care and Use of Laboratory Animals (1996).

[bib27] OECD (2008).

[bib28] Park W.H., Lee S.J., Moon H.I. (2008). Antimalarial activity of a new stilbene glycoside from *Parthenocissus tricuspidata* in mice. Antimicrob. Agents Chemother..

[bib29] Peters W. (1965). Drug resistance in *Plasmodium berghei*. I. Chloroquine resistance. Exp. Parasitol..

[bib30] Ryley J.F., Peters W. (1970). The antimalarial activity of some quinolone esters. Ann. Trop. Med. Parasitology.

[bib31] Sayed N.A., Sukkary M.E., Aiad A., El-Azab W. (2010). Fragmentation mechanism of alkyl-monoglycosides by mass spectrometry. Tenside surf. Det.

[bib32] Slack R.D., Mott B.T., Woodard L.E., Tripathi A., Sullivan D., Nenortas E., Girdwood S.C., Shapiro T.A., Posner G.H. (2012). Malaria-infected mice are completely cured by one 6 mg/kg oral dose of a new monomeric trioxane sulfide combined with mefloquine. J. Med. Chem..

[bib33] Snow R.W., Sartorius B., Kyalo D., Maina J., Amratia P., Mundia C.W., Bejon P., Noor A.M. (2017). The prevalence of *Plasmodium falciparum* in sub-Saharan Africa since 1900. Nature.

[bib34] Soares R.R., da Silva J.M.F., Carlos B.C., da Fonseca C.C., de Souza L.S.A., Lopes F.V., de Paula Dias R.M., Moreira P.O.L., Abramo C., Viana G.H.R., de Pila Varotti F. (2015). New quinoline derivatives demonstrate a promising antimalarial activity against *Plasmodium falciparum in vitro* and Plasmodium berghei *in vivo*. Bioorg. Med. Chem. Lett.

[bib35] Tobinaga S., Sharma M.K., Aalbersberg W.G., Watanabe K., Iguchi K., Narui K., Sasatsu M., Waki S. (2009). Isolation and identification of a potent antimalarial and antibacterial polyacetylene from *Bidens pilosa*. Planta Med..

[bib36] Tostes J.B., Carvalho A.L., Ribeiro da Silva A.J., Mourão P.J.P., Rossi Á.D., Tanuri A., Siani A.C. (2021). Phorbol esters from the latex of *Euphorbia umbellata*: bioguided isolation of highly potent HIV-1 latency interrupters in virus reservoir cells. J. Nat. Prod..

[bib37] Traeger J.C., Morton T.H. (2004). Photoionization of 2, 3-dimethyl-2-butanol (thexyl alcohol): interaction between the charged and expelled fragments. J. Am. Soc. Mass Spectrom..

[bib38] Uwimana A., Legrand E., Stokes B.H., Ndikumana J.L.M., Warsame M., Umulisa N., Ngamije D., Munyaneza T., Mazarati J.B., Munguti K., Campagne P. (2020). Emergence and clonal expansion of *in vitro* artemisinin-resistant *Plasmodium falciparum* kelch13 R561H mutant parasites in Rwanda. Nat. Med.

[bib39] WHO (2021).

[bib40] Yoon I.S., Cho S.S. (2021). Effects of lobetyolin on xanthine oxidase activity *in vitro* and *in vivo*: weak and mixed inhibition. Nat. Prod. Res..

[bib41] Zheng Q., Wang Y., Zhang S. (2021). Beyond alkaloids: novel bioactive natural products from *Lobelia* species. Front. Pharmacol..

