# Supplementary material for: Identification of lobetyolin as a major antimalarial constituent of the roots of Lobelia giberroa Hemsl
Source: Int J Parasitol Drugs Drug Resist. 2022 Jan 22;18:43–51. doi: 10.1016/j.ijpddr.2022.01.002 (PMC8802882; doi:10.1016/j.ijpddr.2022.01.002)
Supplement: Multimedia component 1 [file mmc1.docx]

**Identification of lobetyolin** **as a major antimalarial constituent of the roots of *Lobelia giberroa* Hemsl*.***

Getnet Tadege, Yonatan Alebachew, Ariaya Hymete, Solomon Tadesse*

Department of Pharmaceutical Chemistry and Pharmacognosy, School of Pharmacy, College of Health Sciences, Addis Ababa University, Ethiopia

^*^Corresponding author

Tel.: +251911488473; e-mail: [solomon.tadesse@aau.edu.et](mailto:solomon.tadesse@aau.edu.et)

**Fig. S1.** The HPLC chromatogram of LGF-2.


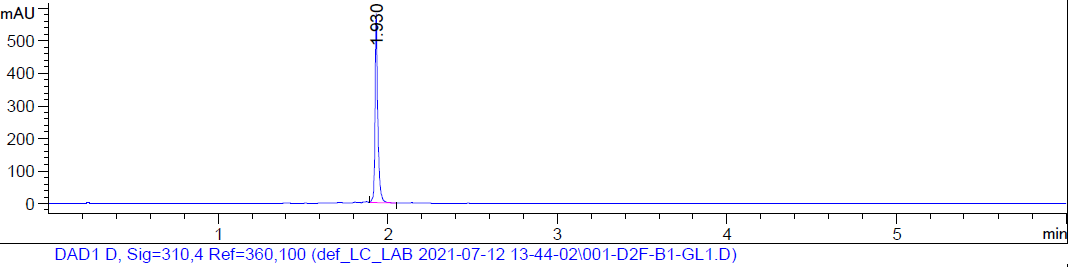


**Fig. S2.** The ^13^C NMR spectrum of LGF-2.

**Fig. S3.** The DEPT-135 spectrum of LGF-2.

**Fig. S4.** The DEPT-90 spectrum of LGF-2.

**Fig. S5.** The ^1^H NMR spectrum of LGF-2.

**Fig. S6.** The HMBC spectrum of LGF-2.


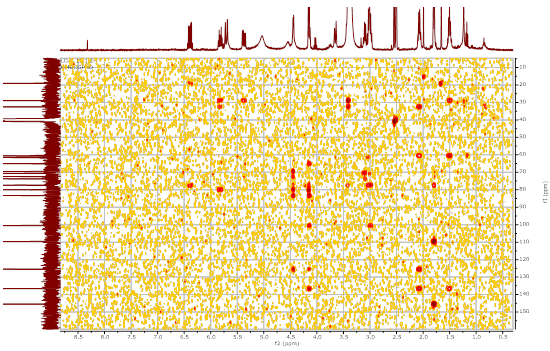


**Fig. S7.** The ^1^H-^1^H COSY spectrum of LGF-2.


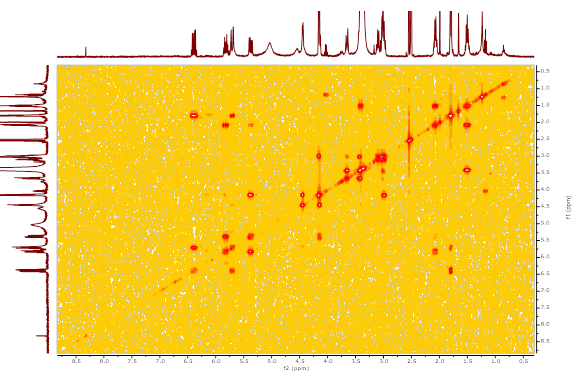


**Fig. S8.** The mass spectrum of LGF-2.


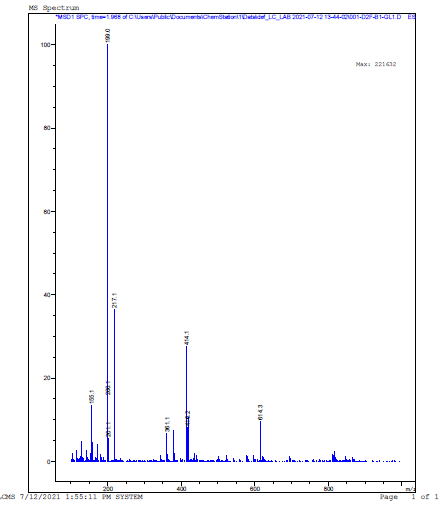


**Fig. S9.** The IR spectrum of LGF-2.


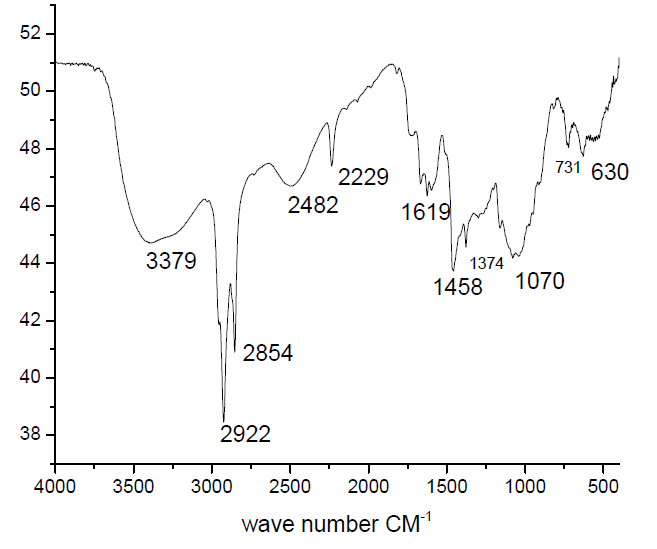


**Table S1.** Body weight, rectal temperature and packed cell volume of *Plasmodium berghei* infected mice before and after administration of the solvent fractions of the roots of *Lobelia giberroa* in the four-day suppressive test.

| Group | Dose (mg/kg) | Weight | | | Temperature | | | Packed cell volume | | |
| --- | --- | --- | --- | --- | --- | --- | --- | --- | --- | --- |
|  |  | D0 | D4 | %Change | D0 | D4 | %Change | D0 | D4 | %Change |
| TW80 |  | 26.96±0.24 | 21.10±0.29 | -21.73±0.91 | 37.10±0.13 | 32.72±0.15 | -11.80±0.55 | 56.5±0.63 | 45.70±0.83 | -19.13±0.66 |
| CQ | 25 | 26.68±0.59 | 26.98±0.64 | 1.11±0.18^a3^ | 37.08±0.06 | 36.90±0.07 | -0.48±010^a3^ | 54.28±0.99 | 53.41±0.89 | -1.59±0.41^a3^ |
| HF | 100 | 27.14±0.75 | 22.46±0.59 | -17.22±0.52^a3b3d3e3^ | 37.02±0.08 | 33.44±0.19 | -9.7±0.41^a1b3e3^ | 55.50±0.83 | 47.94±0.41 | -13.58±0.90^a3b3d3e3^ |
|  | 200 | 26.18±0.73 | 22.82±0.68 | -12.84±0.35^a3b3e2^ | 37.14±0.11 | 33.90±0.17 | -8.72±0.33^a3b3e2^ | 55.06±1.06 | 50.32±0.86 | -8.59±0.23^a3b3e3^ |
|  | 400 | 25.50±0.86 | 23.10±0.0.78 | -9.40±0.42^a3b3^ | 36.94±0.11 | 34.76±0.24 | -5.90±0.60^a3b3^ | 55.88±0.82 | 53.36±0.73 | -4.5±0.14^a3b2^ |
| TW80 |  | 26.96±0.24 | 22.54±0.67 | -14.34±1.34 | 36.9±0.13 | 33.46±0.33 | -9.32±0.86 | 53.92±1.04 | 47.34±0.68 | -1 2.20±0.75 |
| CQ | 25 | 26.68±0.59 | 28.26±0.62 | 5.93±0.4^a3^ | 36.94±0.1 | 36.78±0.12 | -4.34±0.07^a3^ | 53.16±0.49 | 52.97±0.49 | -0.35±0.07^a3^ |
| EAF | 100 | 26.41±0.62 | 24.36±0.88 | -7.84±1.54^a2b3^ | 36.86±0.13 | 35.22±0.16 | -4.45±0.4^a3b3^ | 56.34±0.71 | 52.19±0.91 | -7.37±0.59^a3b3e3d1^ |
|  | 200 | 26.3±0.59 | 23.8±0.72 | -.9.53±1.24^b3e1^ | 36.9±0.14 | 35.22±0.31 | -4.56±0.59^a3b3^ | 55.06±0.49 | 52.42±0.70 | -4.79±0.57^a3b3^ |
|  | 400 | 26.12±1.00 | 24.9±0.77 | -4.54±0.87^a3b3^ | 36.74±0.15 | 35±0.28 | -4.73±0.64^a3b3^ | 54.62±0.55 | 53.02±0.49 | -2.92±0.41^a3b1^ |
| TW80 |  | 26.52±0.39 | 21.82±0.42 | -17.72±1.09 | 37.08±0.73 | 33.42±0.92 | -9.87±0.23 | 56.18±0.82 | 49.92±0.70 | -11.13±0.49 |
| CQ | 25 | 26.92±0.64 | 27.30±0.69 | 1.41±0.42^a3^ | 37.14±0.07 | 36.90±0.07 | -0.64±0.06^a3^ | 55.40±0.93 | 55.08±0.92 | -0.58±0.13^a3^ |
| MF | 100 | 26.68±0.65 | 22.80±0.58 | -14.54±0.38^b3e3d1^ | 36.80±0.08 | 34.31 ±0.16 | -7.52±0.78^a3b3d2e3^ | 55.50±1.07 | 51.36±1.40 | -7.52±0.78^a3b3e3^ |
|  | 200 | 26.40±0.78 | 23.60±0.80 | -10.63±0.89^a3b3e3^ | 37.04±0.08 | 35.36±0.19 | -5.59±0.15^a3b3e3^ | 55.35±0.79 | 52.26±0.83 | -5.59±0.15^a3b3e1^ |
|  | 400 | 27.28±0.71 | 25.94±0.98 | -4.91±0.99^a3b3^ | 36.88±0.12 | 36.26±0.28 | -3.16±0.54^a3^ | 54.48±1.15 | 52.76±1.14 | -3.16±0.54^a3b1^ |
| DW |  | 26.82±0.36 | 21.30±0.42 | -20.59±0.91 | 37.1±0.08 | 32.88±0.24 | -11.37±0.58 | 56.86±0.58 | 49.54±1.17 | -12.92±1.23 |
| CQ | 25 | 26.70±0.45 | 26.82±0.44 | 0.45±0.08^a3^ | 36.88±0.12 | 36.68±0.12 | -0.54±0.09^a3^ | 53.30±1.00 | 52.86±0.95 | -0.81±0.10^a3^ |
| AF | 100 | 26.44±0.64 | 21.78±0.48 | -17.6±0.71^b3d3e3^ | 37.02±0.80 | 33.88±0.17 | -8.48±0.46^a3b3e3d1^ | 55.90±0.63 | 51.69±0.73 | -7.53±0.68^a3b3e2^ |
|  | 200 | 25.78±0.73 | 22.8±0.77 | -11.58±1.10^a3b3c3e3^ | 37.08±0.07 | 34.88±0.59 | -5.93±0.27^a3b3e1^ | 54.38±0.82 | 51.75±0.91 | -4.84±0.59^a3^ |
|  | 400 | 26.26±0.56 | 25.36±0.48 | -3.4±0.44^a3b1^ | 36.92±0.15 | 35.42±0.19 | -4.06±0.35^a3b3^ | 55.32±0.57 | 53.72±0.76 | -2.89±0.76^a3^ |

Data are expressed as mean ± SEM; n = 5; a, compared to negative control; b, to CQ25 mg/kg; c, to 100 mg/kg; d, to 200 mg/kg; e, to 400 mg/kg; 1, p < 0.05; 2, p <0.01; 3, p <0.001; TW80, 2% Tween80; CQ, chloroquine; HF, Hexane fraction; EAF, ethyl acetate fraction; MF, methanol fraction; AF, aqueous fraction; DW, distilled water as a vehicle; D0, pre-treatment value on day 0; D4, post- treatment value on day 4.

**Table S2.** Body weight, rectal temperature and packed cell volume of *Plasmodium berghei* infected mice before and after administration of the column sub-fractions of the roots of *Lobelia giberroa* in the four-day suppressive test.

| Group | Dose mg/kg | Weight | | | Temperature | | | Packed cell volume | | |
| --- | --- | --- | --- | --- | --- | --- | --- | --- | --- | --- |
|  |  | D0 | D4 | %Change | D0 | D4 | %Change | D0 | D4 | %Change |
| TW80 |  | 26.60±0.46 | 23.52±0.50 | -11.57±0.18 | 36.86±0.07 | 33.56±0.29 | -8.95±0.78 | 54.80±0.72 | 47.78±0.51 | -12.79±0.36 |
| CQ | 25 | 26.63±0.64 | 26.75±0.65 | 0.43±0.09^a3^ | 36.92±0.12 | 36.74±0.13 | -0.49±0.13^a2^ | 56.21±0.51 | 56.03±0.52 | -0.32±0.16^a3b3^ |
| LGF-1 | 25 | 26.73±0.55 | 24.12±0.64 | -9.83±0.76^b3^ | 37.04±0.08 | 34.38±0.37 | -7.19±0.83^b3^ | 56.07±0.20 | 51.30±0.37 | -8.50±0.43^a3b3^ |
|  | 50 | 25.16±0.61 | 23.13±0.55 | -8.07±0.61^b3a1^ | 36.84±0.09 | 34.22±0.39 | -7.11±0.01^b3^ | 55.89±0.75 | 51.54±0.95 | -7.81±0.75^a3b3^ |
|  | 100 | 26.87±0.36 | 24.88±0.39 | -7.44±0.40^b3a2^ | 37.12±0.09 | 35.72±0.49 | -3.77±0.37 | 55.18±0.51 | 51.36±0.39 | -6.89±0.04^a3b3^ |
| TW80 |  | 25.86±0.49 | 21.50±0.44 | -16.86±0.71 | 36.70±0.19 | 32.84±0.17 | -10.51±0.40 | 54.76±0.41 | 46.88±0.50 | -14.38±0.96 |
| CQ | 25 | 25.60±0.52 | 26.36±0.59 | 2.95±0.50^a3^ | 36.72±0.09 | 36.54±0.09 | -0.49±0.13^a3^ | 54.04±0.79 | 53.70±0.82 | -0.6321±0.12^a3^ |
| LGF-2 | 25 | 26.14 ±0.99 | 23.12±0.97 | -11.61±0.39^a3b3d3e3^ | 37.02±0.14 | 34.00±0.14 | -8.16±0.15^a3b3e3^ | 54.20±0.98 | 48.32±0.84 | -10.84±0.19^a2b3d1e3^ |
|  | 50 | 24.88±0.79 | 23.10±0.65 | -7.11±0.59^a3b3e3^ | 36.72±0.10 | 34.20±0.20 | -6.86±0.41^a3b3e3^ | 54.98±0.83 | 50.57±0.79 | -8.02±0.32^a3b3e3^ |
|  | 100 | 26.90±0.35 | 26.14±0.39 | -2.83±0.61^a3b3^ | 36.98±0.11 | 35.64±0.07 | -3.62±0.33^a3b3^ | 54.10±0.60 | 51.84±0.79 | -4.19±0.59^a3b3^ |
| TW80 |  | 26.82±0.36 | 21.30±0.42 | -20.59±0.91 | 37.1±0.08 | 32.88±0.24 | -11.37±0.58 | 56.86±0.58 | 49.54±1.17 | -12.92±1.23 |
| CQ | 25 | 26.70±0.45 | 26.82±0.44 | 0.45±0.08^a3^ | 36.88±0.12 | 36.68±0.12 | -0.54±0.09^a3^ | 53.30±1.00 | 52.86±0.95 | -0.81±0.10^a3^ |
| LGF-3 | 25 | 26.44±0.64 | 21.78±0.48 | -17.6±0.71^b3d3e3^ | 37.02±0.80 | 33.88±0.17 | -8.48±0.46^a3b3e3d1^ | 55.90±0.63 | 51.69±0.73 | -7.53±0.68^a3b3e2^ |
|  | 50 | 25.78±0.73 | 22.8±0.77 | -11.58±1.10^a3b3c3e3^ | 37.08±0.07 | 34.88±0.59 | -5.93±0.27^a3b3e1^ | 54.38±0.82 | 51.75±0.91 | -4.84±0.59^a3b3^ |
|  | 100 | 26.26±0.56 | 25.36±0.48 | -3.4±0.44^a3b1^ | 36.92±0.15 | 35.42±0.19 | -4.06±0.35^a3b3^ | 55.32±0.57 | 53.72±0.76 | -2.89±0.76^a3b3^ |

Data are expressed as mean ±SEM; n=5; a, compared to negative control; b, to CQ25 mg/kg; c, to 25 mg/kg; d, to 50 mg/kg; e, to 100 mg/kg; 1, p<0.05; 2, p<0.01; 3, p<0.001; 2% TW80, 2% Tween 80; CQ, chloroquine; LGF-3, *Lobelia giberroa* fraction 3 from column; D0, pre-treatment value on day 0; D4, post- treatment value on day 4

**Table S3.** Body weight, rectal temperature and packed cell volume of *Plasmodium berghei* infected mice before and after administration of lobetyolin in the Rane’s test.

| Group | Dose (mg/kg) | Weight | | | Rectal temperature | | | Packed cell volume | | |
| --- | --- | --- | --- | --- | --- | --- | --- | --- | --- | --- |
|  |  | D0 | D7 | % Change | D0 | D7 | % Change | D0 | D7 | % Change |
| TW80 |  | 26.56±0.57 | 23.42±0.48 | -11.79±0.75 | 36.80±0.17 | 33.62±0.19 | -8.64±0.28 | 54.33±0.62 | 48.92±0.33 | -9.94±0.53 |
| CQ | 25 | 25.80±0.64 | 26.18±0.60 | 1.49±0.12^a3^ | 37.02±0.09 | 36.74±0.11 | -0.75±0.16^a3^ | 53.95±0.51 | 53.60±0.54 | -0.65±0.08^a3^ |
| LBT | 25 | 25.94±0.36 | 24.02±0.41 | -7.42±0.45^a3b3d1e2^ | 36.78±0.16 | 34.20±0.18 | -7.01±0.22^a1b3e3^ | 53.10±0.71 | 49.60±0.48 | -6.57±0.49^a3b3e3^ |
|  | 50 | 25.52±0.69 | 24.23±0.73 | -5.05±0.98^a3b3^ | 36.80±0.12 | 34.64±0.26 | -5.87±0.54^a3b3e2^ | 53.85±0.49 | 50.36±0.43 | -6.47±0.72^a3b3e2^ |
|  | 100 | 27.30±0.33 | 26.24±0.17 | -3.85±0.77^a3b3^ | 36.92±0.15 | 35.60±0.23 | -3.58±0.40^a3b3^ | 52.11±0.69 | 50.34±0.63 | -3.39±0.18^a3b2^ |

Data are expressed as mean ±SEM; n=5; a, compared to negative control; b, to CQ25 mg/kg; c, to 25 mg/kg; d, to 50 mg/kg; e, to 100 mg/kg; 1, p<0.05; 2, p<0.01; 3, p<0.001; 2% TW80, 2% Tween 80; CQ, chloroquine; LBT, *lobetyolin*; D0, pre-treatment value on day 0; D4, post- treatment value on day 4.

**Table S4.** Body weight, rectal temperature and packed cell volume of *Plasmodium berghei* infected mice before and after administration of lobetyolin in the prophylactic test.

| Groups | Dose mg/kg | Body weight | | | Rectal temperature | | | packed cell volume | | |
| --- | --- | --- | --- | --- | --- | --- | --- | --- | --- | --- |
|  |  | D0 | D3 | % Change | D0 | D3 | % Change | D0 | D3 | %Change |
| TW80 |  | 27.45±0.40 | 26.45±0.39 | -2.91 | 36.17±0.14 | 35.23±0.14 | -2.60 | 58.56±0.38 | 56.49±0.38 | -3.53 |
| CQ | 25 | 26.78±0.38 | 26.85±0.38 | 0.26^a3^ | 35.74±0.22 | 35.65±0.20 | -0.25 | 57.70±0.65 | 57.56±0.67 | -0.24^a3^ |
| LBT | 25 | 25.83±0.38 | 25.19±0.38 | -2.48^b3^ | 37.10±0.12 | 36.34±0.11 | -2.48^b3d2e2^ | 57.65±0.49 | 56.30±0.48 | -2.34^a3b3d1e2^ |
|  | 50 | 26.01±0.34 | 25.47±0.35 | -2.08^a1b3^ | 36.80±0.14 | 36.24±0.13 | -1.52^a3b3^ | 56.10±0.49 | 54.93±0.47 | -2.08^a3b3^ |
|  | 100 | 27.86±0.55 | 27.00±0.53 | -1.47^a2b3^ | 36.75±0.13 | 36.40±0.13 | -0.95^a3b3^ | 56.73±0.66 | 55.66±0.67 | -1.88^a3b3^ |

Data are expressed as mean ±SEM; n=5; a, compared to negative control; b, to CQ25 mg/kg; c, to 25 mg/kg; d, to 50 mg/kg; e, to 100 mg/kg; 1, p<0.05; 2, p<0.01; 3, p<0.001; 2% TW80, 2% Tween 80; CQ, chloroquine; LGF3, *L. giberroa* fraction 3 from column; D0, pre-treatment value on day 0; D4, post- treatment value on day 4.
